# “We Are Young, We Run Free”: Predicting Factors of Life Satisfaction among Young Backpackers

**DOI:** 10.3390/ijerph19031429

**Published:** 2022-01-27

**Authors:** Tehila Refaeli, Shlomit Weiss-Dagan, Drorit Levy, Haya Itzhaky

**Affiliations:** 1 The Charlotte Jack Spitzer Department of Social Work, Ben-Gurion University of the Negev, Beer Sheva 8499000, Israel; 2 Louis and Gabi Weisfeld School of Social Work, Bar-Ilan University, Ramat-Gan 5290002, Israel; shlomit.weiss-dagan@biu.ac.il (S.W.-D.); Drorit.levy@biu.ac.il (D.L.); haya.itzhaky@biu.ac.il (H.I.)

**Keywords:** backpackers, life satisfaction, risk-taking behaviors, personal resources, social support, community participation, mediation

## Abstract

Although research from a positive psychology perspective is conducted among different populations, few studies have examined the predictors of life satisfaction among young backpackers. The current study focused on young adults (ages 21–30), an age group for whom backpacking treks are a growing phenomenon, during their treks in the Far East and South America. Direct and indirect models were used to identify personal factors and environmental resources contributing to life satisfaction. After at least one month abroad, 318 young adults (M = 23.76) answered a self-report quantitative questionnaire. The findings show that personal resources, social support, and community participation were positively associated with life satisfaction, and risk-taking behaviors were negatively associated with life satisfaction. Social support and community participation partially mediated the association between risk-taking behaviors and life satisfaction and between personal resources and life satisfaction. The implications of the findings for the subjective well-being of young backpackers during their transition to adulthood include, among others, the need to help young backpackers maintain their personal and social resources as valuable assets for coping with challenges during their trips. It is also important to increase awareness of the possible wide-ranging negative effects of risk-taking behaviors during backpacking trips.

## 1. Introduction

Backpacking trips have become a rite of passage for many young people prior to settling into responsible, adult lives. Generally, these trips can only be afforded by young people of higher economic classes [1]. Backpacking is referred to as a specific form of tourism [2,3], and backpackers as having specific characteristics, such as being young people who tend to stay in budget accommodations, seek contact with other travelers, are flexible in their travel plans and schedules, and usually stay for longer periods than “regular” tourists would [3,4,5,6]. For some young people, the backpacking trip is part of a gap year before they commit to adult responsibilities [7].

Most backpackers take these trips during emerging adulthood, which is the developmental period that extends between the ages of 18 and 29 [8], a time when many young people remain dependent on their parents’ financial, concrete, and emotional support. During this period, emerging adults’ main developmental tasks involve identity formation and making decisions about their futures, tasks that they accomplish in part through an exploration of their options [8,9]. For some young people, the backpacking trip constitutes an important part of the process of self-exploration that they undertake to discover what they would like to be and do in their “real” adult lives [10,11].

Many studies have focused on life satisfaction among young people who are students, e.g., [12,13], but to the best of our knowledge, life satisfaction among other groups of young adults, and specifically among backpackers, has scarcely been examined. Although backpacking is a leisure activity and would therefore be expected to positively impact life satisfaction, it includes several factors that can negatively contribute to life satisfaction—for instance, being far from familiar support networks, or engaging in risky behaviors (ideas which will be elaborated upon below).

Therefore, and in light of the significant growth in the backpacking trend witnessed among young people in recent years, the aim of this study was to explore young adults’ life satisfaction and the factors that might predict such satisfaction in the context of backpacking trips, when these young people are far away from the comforts of home, family, and community. More specifically, the research questions were (1) whether personal resources (sense of mastery and self-esteem) and environmental resources (social support and community participation) are associated with life satisfaction and (2) whether environmental resources mediate the personal resources–life satisfaction association.

### 1.1. Subjective Well-Being and Life Satisfaction

Life satisfaction is a component of subjective well-being (SWB), which, in some of the literature, is used interchangeably with SWB [14,15]. Diener and associates [16] defined SWB as people’s cognitive and affective evaluation of their lives as a whole. As such, the level of life satisfaction may reflect the gaps between people’s desired and actual accomplishments in life [17] and is equally applicable to a specific life domain (work, marriage, family, etc.) and to people’s general evaluation of their lives [18,19].

In the current study, we used Hobfoll’s theory of conservation of resources (COR) [20] to elucidate the risk and protective factors predicting life satisfaction among young adults backpacking in the Far East and South America. Whereas Hobfoll’s theory [20] refers to the importance of personal resources and the possible contribution of social support to life satisfaction, here we assessed life satisfaction from a wider perspective that also incorporated the influences of risk-taking behaviors and community resources. The conservation of resources theory suggests that individuals who possess few resources are more vulnerable to the loss of resources, whereas those who already have more resources are able to gain even more [21]. Accordingly, we examined the mediating effects that environmental resources (i.e., social support and community participation) have on the associations between risk-taking behaviors and life satisfaction and between personal resources and life satisfaction among young backpackers.

In regard to backpacking and life satisfaction, two other theories should also be mentioned. The activity theory suggests an association between the frequency of leisure activity and the life satisfaction level [22]. The need theory, for its part, highlights the notion that when individuals’ needs are gratified, their life satisfaction improves [23]. Although both theories have been used to explain the association between leisure activities and life satisfaction, they do not fully explain what contributes to life satisfaction [24]. Namely, although backpacking may contribute to young peoples’ life satisfaction by way of the frequency of such trips (or their length) or by fulfilling their needs, or both, other factors can also play a role in their life satisfaction in this context. In the current study, therefore, we explored other factors that might contribute to young backpackers’ life satisfaction.

### 1.2. Risk-Taking Behaviors

Risk-taking behaviors entail a wide range of activities with potentially negative or dangerous consequences for the person who engages in them and/or for other people, and the nature of these behaviors varies according to the context in which they are carried out [25]. Such behaviors, which can be manifested in sensation-seeking, risk-taking, or reckless behavior, are highly relevant to young backpackers [26,27,28]. Earlier studies have indicated that this group tends to exhibit higher rates of exposure to risk-taking behaviors, including sex with casual partners, unprotected sex, reckless driving, and substance use, to provide only a few examples, e.g., [27,28,29,30,31]. Although some studies have investigated how the characteristics of the emerging adulthood period are related to risk-taking behavior [32,33], we could not locate any discourse on this issue in the literature, specifically in terms of backpacking, a phenomenon which mainly exists during emerging adulthood. Many studies among adolescents and students have indicated the positive effect of life satisfaction on limiting risk-taking behaviors [34,35]. Risk-taking behaviors and their implications have also been examined in terms of life satisfaction among adolescents and college students, with studies indicating an inverse relationship between risk-taking behavior and life satisfaction [36,37,38]. Baláž and Valuš [39] suggested that when people take risks, they understand that undesired consequences may ensue, but they also know there is the possibility for positive outcomes. As such, based on the need theory [23], it could be posited that risk-taking behavior may enhance backpackers’ life satisfaction.

### 1.3. Personal Resources

Personal resources, such as a sense of mastery and self-esteem, constitute a person’s psychological capital. *Sense of mastery* refers to the perceived control that individuals have over their futures, their perceived ability to influence important aspects in their lives, and their perceived ability to cope with life stressors [40,41]. *Self-esteem* refers to the individual’s self-image, which is based on self-evaluations about appearance, aspirations, and the ability to cope with challenges [41,42]. Although many studies have shown that self-esteem is positively associated with life satisfaction, e.g., [43,44,45,46], few have examined the association between a sense of mastery and life satisfaction in different populations [47,48] and specifically among adolescents [49,50]. One study conducted among young adults did indicate that proactive coping, a variable that is similar to a sense of mastery, was related to life satisfaction [51]. That said, the contribution of these factors to life satisfaction among backpackers has not been examined.

### 1.4. Environmental Resources

Similar to personal resources, environmental resources can also make a profound contribution to life satisfaction. *Social support* is defined as the exchange of resources between at least two people with the aim of improving the financial, concrete, or emotional standing of one of them [52]. Among young backpackers, social support may manifest in a distinct fashion. As they are far from their home networks, including their families and peers, some backpackers will travel in groups of pre-existing friends, whereas others will form new, supportive relationships during their journeys [53,54,55]. Studies have examined social support’s contribution, whether from parents, family members, and/or peers, to life satisfaction among different groups of young people during this critical life stage [56,57,58,59]. However, we could not find any studies that examined this issue among backpackers. In light of the contribution that social support makes to life satisfaction [56,58,60,61], in this study, we explored the direct effect of social support on life satisfaction. Based on the COR theory [20,21] and on earlier studies among other populations, e.g., [61,62,63], we also explored the mediating effect that social support has on life satisfaction.

Another environmental resource, *community participation*, refers to one’s involvement in and feelings of responsibility for the community, including the behavioral dimension of that involvement [64]. Community participation also refers to how citizens affect decision making on issues that are relevant to their desired quality of life [65,66]. Studies have shown that, in general, one’s community participation tends to decrease during the period of emerging adulthood [67,68,69], a finding that is due ostensibly to the egoistic nature of this period of human development [70]. This outcome, however, may vary across cultures [71]. Although the contribution of community variables to life satisfaction has been widely examined in adult populations, e.g., [72,73,74,75], it has not been examined among young backpackers. In the current study, therefore, we examined the direct and mediating effects of community participation vis à vis life satisfaction.

In sum, our research model was based on the COR theory [20,21], which refers to the risk and protective factors predicting life satisfaction. Previous studies have explored the cumulative contribution of some of the variables included in this study to the prediction of life satisfaction [63,75,76]. Some have also explored mediating models [63,75]. That said, most of these studies did not focus on young adults, or specifically on backpackers. As such, in this study we aimed to incorporate both personal and environmental factors into one comprehensive model in predicting life satisfaction among backpackers.

### 1.5. The Study Goals

This study presents data about predictors of life satisfaction among young adult backpackers traveling abroad, a population rarely examined in this context. On the one hand, these young adults might experience backpacking as a type of leisure activity that can have a positive impact on life satisfaction [51]. On the other hand, they may feel burdened by the stress of being far away from their parents and familiar surroundings [77], important sources of support during the challenging transition-to-adulthood phase of life. Therefore, exploring factors contributing to life satisfaction during young adults’ backpacking treks will potentially contribute to, and widen, the existing literature on life satisfaction and SWB.

Based on the COR theory [20,21], the study first tested a predictive model for the life satisfaction of young backpackers during their journeys based on their risk-taking behaviors and on the personal resources (sense of mastery and self-esteem) and environmental resources (social support and community participation) at their disposal. In addition, in this study we explored mediating models for the associations between risk-taking behaviors and personal resources on the one hand and life satisfaction on the other, in which environmental resources functioned as mediators.

## 2. Materials and Methods

### 2.1. Participants

The participants in this study were young Israeli backpackers between the ages of 21 and 30 with an average age of 23.76 (SD 1.56) and a median age of 23, who had been traveling in India, Nepal, and South America for at least one month. Our primary sample included 387 young people. Sixty-five of them were removed due to having completed fewer than half of the questionnaire items, and four were removed as they were outliers and affected the normal distribution of the data. Our final sample thus comprised 318 young adults. The descriptive statistics of the sample are shown in Table 1. More than half of the participants were women. The majority of the participants were born in Israel, and their fathers were also native Israelis. More than 2/3 of the participants were secular, and 90% of the participants had high school diplomas.

The average stay abroad of the participants was 3.48 months (SD 2.90), with a median of three months. Over half of them had been in South America, more than a fifth in India, and about a fifth in Nepal.

### 2.2. Procedure

Data were collected between April 2014 and March 2016, and convenience sampling was used to collect the data. One of the researchers traveled to the Far East (India and Nepal) and to South America (Chile, Uruguay, Paraguay, Peru, Argentina, and Brazil) to ensure that we would obtain an acceptable questionnaire response rate (see Appendix A with the participants’ locations). The abovementioned researcher approached all of the young travelers she met personally to ask them to answer the questionnaire. On a daily basis, she visited their guesthouses, the restaurants where they ate, and any other places where they usually met, in order to recruit people. The questionnaire was administered in electronic form only (computer, phone, or tablet), and a link was sent to participants by the researcher via email or WhatsApp. Roughly 5% of the participants filled out the questionnaire on Facebook. Completing the questionnaire took on average about 30 min. There is no way to determine exactly how many backpackers received the link and did not respond to it.

### 2.3. Measures

*Demographic characteristics*. Participants were asked to provide data about gender, year of birth, country of birth, their father’s country of birth, level of education, the duration of the trip abroad, and place of residence at the time of the research.

The entire questionnaire consisted of several widely used scales (see also Appendix B):

*Life satisfaction*. We used the Student’s Life Satisfaction Scale developed by Huebner [78], which comprises seven items that examine general life satisfaction (e.g., “My life is good”). Responses were graded by a four-point scale ranging from 1 (never) to 4 (almost always). Cronbach’s alpha was 0.89.

*Risk-taking behaviors*. This measure was based on a combination of two questionnaires [79,80]. We used only one component from these questionnaires, referring to reports on the frequency of involvement in 13 risk-taking behaviors (i.e., driving, health, breaking the law, and substance abuse) during the last year. Responses were based on a scale ranging from 1 (never) to 4 (almost always)—for example, how often in the last year “you got drunk”. Cronbach’s α was 0.91.

*Sense of mastery*. Questionnaire items were developed by Pearlin and Schooler [42]. Consisting of seven statements, they explore people’s sense of being able to control things in their life, for example, “I have little control over the things that happen to me”. Participants were asked to indicate, on a scale ranging from 1 (strongly agree) to 5 (strongly disagree), the extent to which each statement described them. Cronbach’s alpha was 0.90 in the present study.

*Self-esteem*. Developed by Rosenberg [81], this scale includes 10 items, for example, “On the whole, I am satisfied with myself”. Responses were based on a five-point scale ranging from 1 (strongly agree) to 5 (strongly disagree). The Cronbach’s alpha in the present study was 0.91.

*Social support*. The level of perceived social support was measured via the Multidimensional Scale of Perceived Social Support (MSPSS) questionnaire developed by Zimet, Dahlem, Zimet, and Farley [82]. It contains 12 statements that measure the respondent’s perceptions of support from family, friends, and significant others (e.g., “my family is really trying to help me”). Responses were based on a seven-point scale ranging from 1 (very strongly disagree) to 7 (very strongly agree). The Cronbach’s α in the current study was 0.94.

Community participation. Developed by Itzhaky and York [83], this scale includes four components, such as “I have an influence over my community”. The items were measured on a five-point scale ranging from 1 (do not agree at all) to 5 (strongly agree). The Cronbach’s alpha was 0.80. As they were abroad at the time of the research, participants were asked to refer to their level of community participation prior to having left for their trips.

### 2.4. Data Analysis

To examine the association between sociodemographic characteristics and life satisfaction, t-tests were conducted, after which Pearson correlations were calculated to verify the association between the personal and environmental variables and life satisfaction. A four-step multivariate hierarchical logistic regression was conducted to examine the contributions of risk-taking behaviors and of personal and environmental variables to the prediction of life satisfaction. The assumptions of regression analysis of multivariate normality, absence of multicollinearity, and homoscedasticity were met. Lastly, the PROCESS macro created by Hayes [84] in SPSS (IBM, Chicago, IL, USA) was used to examine the mediating models to determine the associations between risk-taking behaviors and personal resources on the one hand and life satisfaction on the other, in which environmental resources functioned as mediators.

### 2.5. Ethical Considerations

The study adhered to the ethical guidelines of the authors’ respective institutions and was approved by the ethics committee of one of the universities. All the participants signed informed consent, and they were assured that their identities would remain confidential. The questionnaire itself did not include any personally identifying details.

## 3. Results

The *t*-tests for independent samples revealed significant differences in both gender and religious status with regard to life satisfaction. Men (M = 2.93, SD = 0.64) reported higher life satisfaction than women, as determined by the following values: M = 2.74, SD = 0.71, t(302) = 2.44, *p* < 0.05. Religious people (M = 2.67, SD = 0.78) reported lower life satisfaction than secular people, as determined by the following values: M = 2.89, SD = 0.64, t(302) = 2.52, *p* < 0.05. Place of residence at the time of the research was not significantly associated with life satisfaction, for which F(3299) = 0.59; n.s.

To examine the associations that personal and environmental variables had with life satisfaction, we conducted a series of Pearson correlations. These analyses revealed that risk-taking behaviors were negatively correlated with life satisfaction, such that a higher incidence of risk-taking behaviors related to lower life satisfaction (r = −0.43, *p* < 0.001). All of the personal and environmental resources correlated positively with life satisfaction, including a sense of mastery (r = 0.77, *p* < 0.001), self-esteem (r = 0.77, *p* < 0.001), social support (r = 0.66, *p* < 0.001), and community participation (r = 0.52, *p* < 0.001).

### 3.1. Multivariate Prediction of Life Satisfaction

We conducted a four-step multivariate hierarchical regression analysis to explore the combined contribution of risk-taking behaviors and personal and environmental resources to life satisfaction. Overall, the multivariate model accounted for 69.3% of the variance in life satisfaction, where F(7296) = 94.02, *p* < 0.001. As shown in Table 2, risk-taking behaviors and personal and environmental resources contributed to the prediction of life satisfaction after controlling for background variables. In the first step, gender and religious orientation were associated with life satisfaction. Women and religious participants had significantly lower life satisfaction than men and secular young people. Participant background characteristics accounted for 3.7% of the explained variance in life satisfaction. In the second step, risk-taking behaviors correlated negatively with life satisfaction and explained 16.9% of the variance. Regarding the personal resources in the third step, both a sense of mastery and self-esteem were strongly associated with life satisfaction and had the strongest predictive power, accounting for 44.9% of the variance. The fourth step, which added environmental resources, explained a further 3.8% of the variance, as social support and community participation were found to contribute significantly to the explained variance.

### 3.2. Mediation Analyses of the Association between Risk-Taking Behaviors, Personal Resources, and Life Satisfaction

We examined the potential of environmental resources to function as mediators of the associations between risk-taking behaviors, personal resources, and life satisfaction by using the PROCESS macro for SPSS [84]. Bootstrapping was used to evaluate the significance of the mediators. Estimates of the indirect effects were based on running 5000 bootstrap iterations of computed samples on a 95% confidence interval (CI). Table 3 presents the standardized regression coefficients of the direct (c path) and indirect (c′ path) effects. The reduction in the coefficient from the c path to the c′ path and the absence of zero in the 95% confidence intervals indicate significant mediation effects.

A negative direct effect was found between risk-taking behaviors and life satisfaction (b = −0.42). In addition, a significant indirect connection was found via mediation by social support (b = −0.13, 95%CI = −0.376, −0.205). The mediation model was partially supported, as some of the effect of the risk-taking behaviors on life satisfaction was expressed directly. The indirect effect via the mediation by community participation (b = −0.36, 95%CI = −0.127, 0.002), was not significant.

A strong, positive, direct effect was found between mastery and life satisfaction (b = 0.77). In addition, two significant indirect connections, through mediation by social support (b = 0.59, 95%CI = 0.108, 0.253) and by community participation (b = 0.67, 95%CI = 0.057, 0.139), were detected. The mediation models were partially supported, as some effect of mastery on life satisfaction was expressed directly.

Finally, a significant, positive, direct effect was also found between self-esteem and life satisfaction (b = 0.76). In addition, the two indirect connections to life satisfaction that were assessed—via the mediation by social support (b = 0.57, 95%CI = 0.129, 0.255) and via the mediation by community participation (b = 0.67, 95%CI = 0.058, 0.141)—were significant. The mediation models were partially supported, as some effect of self-esteem on life satisfaction was expressed directly.

## 4. Discussion

Backpacking journeys have become popular among young adults around the world but have not adequate research attention. Guided by the positive psychology movement, which focuses on factors that promote quality of life and life satisfaction, the current study focused on untangling the factors that contribute to high life satisfaction among young backpackers. On the basis of the activity theory, which indicates that the frequency or length of a leisure activity is important to life satisfaction [22], and the need theory, which highlights that gratifying needs improves life satisfaction [23], backpacking trips may enhance life satisfaction. However, young backpackers may, alternatively, experience low life satisfaction as a function of their being in the emerging adulthood stage of life. That is, backpacking trips tend to take place at a time when young people need to make decisions about their near futures [10,11]. Additionally, during these trips, young people are far away from their parents and natural social networks [77], which are important contributors to life satisfaction.

Previous studies regarding the association between risk-taking behaviors and life satisfaction among adolescents and young people have yielded mixed findings [36,37,38]. Some of the theoretical literature has indicated the possibility that risk-taking behaviors enhance life satisfaction [23,39]. However, the results of our study clearly indicate that risk-taking behaviors are strong predictors of low life satisfaction among young backpackers. One possible explanation for this outcome is that the young people in the current study were in the midst of backpacking trips in developing countries, a scenario in which the potential and temptation to engage in risky behaviors, such as drug use or unprotected sex, was much higher than in their home countries [27]. As such, young people who chose not to take such risks may have made that choice due to their greater levels of life satisfaction. Likewise, those who did engage in risky behaviors may have subsequently become preoccupied with concerns about the possible ramifications (e.g., health and legal) of their behavior, which, in turn, may have affected their levels of life satisfaction. These findings echo previous findings showing that young people who expose themselves to risky behaviors expect their levels of happiness to decline as they age [85].

Although risk-taking behaviors potentially threaten life satisfaction, people typically have protective factors that function to maintain life satisfaction. Additionally, according to the COR theory, at times of stress people make an effort to protect their SWB via retaining and enhancing their personal resources [20,21]. In line with the results of previous studies among various populations, e.g., [44,46,47,48,50], our findings indicate that personal resources—specifically, self-esteem and sense of mastery—contributed to increased life satisfaction among the young backpackers. Moreover, our results also show that personal resources contributed to social support and community participation, which functioned as mediating variables that, in turn, also promoted increased life satisfaction. Therefore, our findings support the COR theory, indicating that people who already have more resources at their disposal are more able to gain additional resources than people with fewer resources [21].

In this study, the environmental resources of social support and community participation were shown to function as mediating variables that promote life satisfaction. Previous studies have indicated that young Israeli backpackers rely significantly on peer support [53,86]. Additionally, the COR theory emphasizes the benefits of social support when it fulfills situational needs [87]. Thus, social support during young people’s backpacking adventures can fulfill psychological needs such as companionship, which can contribute to life satisfaction. Furthermore, according to Arnett [70], social support can markedly facilitate success in confronting the challenges associated with the transition to adulthood, whereas the lack of such support has been associated with a reduction in overall well-being.

The importance of social support for emerging adults who are on backpacking trips is also reflected in the mediating role that social support plays in the association between risk-taking behaviors and personal resources on the one hand and life satisfaction on the other. Backpacking trips are notorious for the increases in risky behavior they promote as mentioned above and as described in numerous studies regarding the backpacking phenomenon around the world, e.g., [28,29,31]. In this context, it is important to note that risk-taking behavior is correlated with decreased social support, which, in turn, is related to lower life satisfaction. Thus, we can assume that when young people are far removed from the normative rules of their home countries—a scenario in which they tend to take unusual risks—those who intentionally expose themselves to extremely dangerous scenarios experience less support from their peers, support that is especially important to their happiness when they are removed from their familiar surroundings. A previous study supported this notion and found that risk-taking behavior is more common among backpackers who travel alone [88]. On the other hand, peer support during backpacking trips has been found to be a protective factor in terms of risk-taking behaviors [89].

The strong correlation between personal resources and life satisfaction was partially mediated by the effect of social support. Namely, it is easier for young people with strong personal resources to obtain adequate social support even when they are abroad and, as mentioned, this support can promote their life satisfaction. Our findings suggest that because young backpackers are both in a critical transitional period in their lives and also removed from their everyday surroundings, when their familiar network of support changes, they need to exploit their personal resources to develop new supportive relationships. Thus, our results are in line with Hobfoll’s theory [21], which emphasizes that during periods of stress, people who possess more robust personal resources (such as self-esteem and self-efficacy) are more able to maintain and even strengthen their environmental resources (such as social support). Indeed, their stronger resources can help them cope better with stress and can protect their SWB [90].

In the current study, we examined community participation as a unique predictor of life satisfaction among young backpackers. As mentioned, although elements of community participation have been found to be related to life satisfaction for different age groups [72,73,74,75], studies indicate that community participation tends to decrease during emerging adulthood [67,69]. To the best of our knowledge, our study is the first to explore the contribution of community participation to life satisfaction among emerging adults in general and specifically among young backpackers. The findings indicate that community participation has both direct and mediating effects. This result suggests that even during the emerging adulthood period, when young people tend to be more egoistic [70], feelings of belonging and of being part of the community are important to young backpackers’ life satisfaction. A possible explanation for these findings is rooted in the great distance that exists between these young people and their familiar surroundings. Far from home, they may tend to view their participation in the community more positively than how they perceived it when they were in their respective home countries, and therefore their participation may be more significant in predicting their life satisfaction.

Several demographic characteristics were found to be related to life satisfaction. Our study indicated that men were more satisfied with their lives than women. In many previous studies, gender was found to be related to life satisfaction, but the findings have been mixed [91,92]. Other studies conducted among young adults did not find significant gender differences in terms of life satisfaction [58,93,94]. It may be that as a result of the poor living conditions that generally typify backpacking trips, the women participants experienced reduced life satisfaction. Israeli men, however, may be more accustomed to and familiar with such conditions, given their prior military combat service.

In terms of religiosity, previous studies conducted among both young adults [95] and the general population [96,97] have indicated that people who are religious have higher life satisfaction. However, our study indicated a reverse finding, perhaps because the higher life satisfaction of religious people partially derives from the religious social networks and social activities in which they take part [98]. These activities and networks are generally not available for religious backpackers; backpackers may even find it hard to observe religious customs during their backpacking trips, which may negatively affect their well-being.

Our research had several limitations. In this study we did not examine the contribution of any socioeconomic variables to life satisfaction, which, according to previous studies, can affect one’s level of life satisfaction, e.g., [99,100]. That said, most young people who embark on backpacking trips are from medium to high socioeconomic backgrounds, and therefore, they can presumably afford such adventures. Another limitation may have been the effect of culture on issues such as social support and community participation [71]. The significant contributions made by social support and community participation to life satisfaction in this study may have been due to the tendency of Israeli backpackers to travel in groups with other Israelis and actively search for places during their trips where they can meet their compatriots [101]. However, data regarding whether participants were traveling alone, in groups, or with partners were lacking in the current study, and these aspects may have had an impact on participants’ life satisfaction. Lastly, another limitation of this study was the response rate of the participants, many of whom were traveling in developing regions where access to the internet was limited and unstable. Therefore, we could not know whether the lack of response was due to technical constraints or because young people did not wish to participate.

## 5. Conclusions

The results of this study show that different factors can contribute to life satisfaction among emerging adults during backpacking trips abroad. Although personal resources such as sense of mastery and self-esteem seemed to have the strongest effects on life satisfaction, our study also indicated that risk-taking behaviors can jeopardize this group’s life satisfaction and that social support and community participation can function as protective factors. In the framework of the COR theory [20], we would suggest that during the transition to adulthood—a unique developmental period in human life that entails a marked threat to one’s life satisfaction—it is important to help young backpackers maintain their personal and social resources, as these may be valuable assets for successfully coping with the challenges of this period and of their trips.

Our findings suggest directions for future research. As the longitudinal transition to adulthood manifests itself similarly around the world, it is important to further examine whether there are cultural differences in predictors of life satisfaction among young people who take “gap years” or backpacking trips and those who do not concerning their life satisfaction and its predictors. In addition, because the results of this study indicate that personal resources play a significant role for young backpackers, studies should evaluate interventions that focus on empowering young people by helping them maintain and strengthen their personal resources before and during their backpacking trips. Given that the data in this study were collected before the COVID-19 pandemic, it would also be important to examine whether an association exists between the pandemic and/or fear of contagion and life satisfaction of backpackers in different countries. Lastly, future studies should examine the potential contributions of different community resources (such as community sense of belonging, community solidarity, leadership, etc.) in greater depth, as well as volunteer tourism, to life satisfaction among young backpackers.

## Figures and Tables

**Table 1:** Descriptive statistics of the sample (*N* = 318).

Variables	*N*	%/*Mean (SD)*
Age	318	23.76 (1.56)
Gender (female = 1)	176	55.3%
Country of birth (Israel = 1)	297	93.4%
Father’s country of birth (Israel = 1)	248	81.3%
Religiosity (religious = 1)	91	28.6%
Education (high school diplomas = 1)	284	89.6%
Place of stay abroad		
South America	185	58.4%
India	73	23.0%
Nepal	55	17.3%
Average stay abroad	314	3.48 (2.90)

**Table 2:** Contribution of background and personal and environmental variables to explaining life satisfaction: hierarchical multivariate linear Regression.

	*R* ^2^	Δ*R*^2^	*B*	*SE*	*β*
*Step 1*	0.04 **				
Gender ^a^			−0.18	0.06	−0.13 *
Religiosity ^b^			−0.21	0.09	−0.14 *
*Step 2*	0.21 ***	0.17 ***			
Risk-taking behaviors			−0.51	0.06	−0.42 ***
*Step 3*	0.66 ***	0.45 ***			
Sense of mastery			0.32	0.05	0.41 ***
Self-esteem			0.36	0.05	0.42 ***
*Step 4*	0.69 ***	0.04 ***			
Social support			0.11	0.03	0.20 ***
Community participation			0.10	0.03	0.15 ***

* *p* ≤ 0.05; ** *p* ≤ 0.01; *** *p* ≤ 0.001. ^a^ 0 = male; 1 = female. ^b^ 0 = secular; 1 = religious.

**Table 3:** Standardized coefficients of the direct and indirect connections of the independent variables with life satisfaction (5000 bootstraps) and the effects of mediators.

Independent Variable	Mediator	Direct Effect (c Path)	Indirect Effect (c′ Path)	CI
Risk-taking behaviors	Social support	−0.42 ***	−0.13 **	−0.376, −0.205
	Community participation		−0.36 ***	−0.127, 0.002
Sense of mastery	Social support	0.77 ***	0.59 ***	0.108, 0.253
	Community participation		0.67 ***	0.057, 0.139
Self-esteem	Social support	0.76 ***	0.57 ***	0.129, 0.255
	Community participation		0.67 ***	0.058, 0.141

Note: The dependent variable is life satisfaction. Standardized regression coefficients and bias-corrected 95% confidence intervals are displayed. Confidence intervals that do not include zero are statistically significant at *p* < 0.05. ** *p* < 0.01; *** *p* < 0.001.

**Figure A1:**
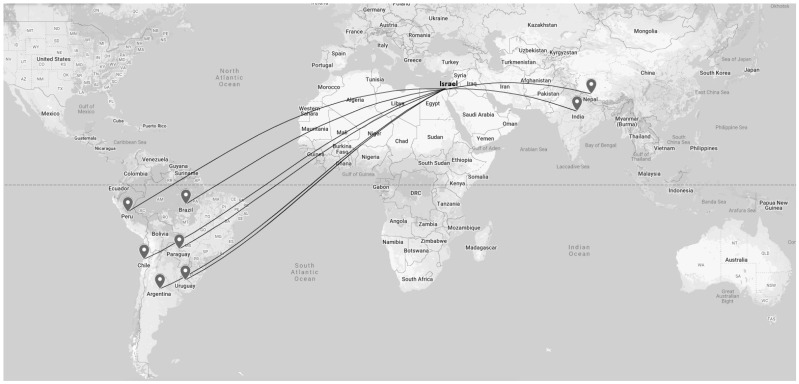
The participants’ locations at the time of the study.

## Data Availability

The data that support the findings of this study are available from the corresponding author upon reasonable request.

## Appendix B

**Table A1:** Scales used in the questionnaire.

Variable	Source	Number of Items	Answers Range
Gender		1	0 = male; 1 = female
Religiosity		1	0 = secular; 1 = religious
Year of birth		1	-
Country of birth		1	0 = Israel; 1 = other
Father’s country of birth		1	0 = Israel; 1 = other
Level of education		1	0 = does not have high school diploma 1 = has high school diploma
Duration of the trip abroad		1	-
Place of residence at the time of the research		1	1-India; 2-Nepal; 3-South America
Life satisfaction	Huebner (1991) [78]	7	1 (never) to 4 (almost always)
Risk-taking behaviors	Shapiro, Siegel, Scovill, and Hays 1998; Siegel et al. 1994 [79]	13	1 (never) to 4 (almost always)
Sense of mastery	Pearlin and Schooler (1978) [42]	7	1 (strongly agree) to 5 (strongly disagree)
Self-esteem	Rosenberg (1965) [81]	10	1 (strongly agree) to 5 (strongly disagree)
Social support	Zimet, Dahlem, Zimet and Farley (1988) [82]	12	1 (very strongly disagree) to 7 (very strongly agree)
Community participation	Itzhaky and York (1994) [83]	4	1 (do not agree at all) to 5 (strongly agree)
